# Calcyclin Binding Protein/Siah-1 Interacting Protein Is a Hsp90 Binding Chaperone

**DOI:** 10.1371/journal.pone.0156507

**Published:** 2016-06-01

**Authors:** Agnieszka Góral, Paweł Bieganowski, Wiktor Prus, Łucja Krzemień-Ojak, Beata Kądziołka, Hanna Fabczak, Anna Filipek

**Affiliations:** 1 Nencki Institute of Experimental Biology PAS, Warsaw, Poland; 2 Mossakowski Medical Research Centre PAS, Warsaw, Poland; University of Geneva, SWITZERLAND

## Abstract

The Hsp90 chaperone activity is tightly regulated by interaction with many co-chaperones. Since CacyBP/SIP shares some sequence homology with a known Hsp90 co-chaperone, Sgt1, in this work we performed a set of experiments in order to verify whether CacyBP/SIP can interact with Hsp90. By applying the immunoprecipitation assay we have found that CacyBP/SIP binds to Hsp90 and that the middle (M) domain of Hsp90 is responsible for this binding. Furthermore, the proximity ligation assay (PLA) performed on HEp-2 cells has shown that the CacyBP/SIP-Hsp90 complexes are mainly localized in the cytoplasm of these cells. Using purified proteins and applying an ELISA we have shown that Hsp90 interacts directly with CacyBP/SIP and that the latter protein does not compete with Sgt1 for the binding to Hsp90. Moreover, inhibitors of Hsp90 do not perturb CacyBP/SIP-Hsp90 binding. Luciferase renaturation assay and citrate synthase aggregation assay with the use of recombinant proteins have revealed that CacyBP/SIP exhibits chaperone properties. Also, CacyBP/SIP-3xFLAG expression in HEp-2 cells results in the appearance of more basic Hsp90 forms in 2D electrophoresis, which may indicate that CacyBP/SIP dephosphorylates Hsp90. Altogether, the obtained results suggest that CacyBP/SIP is involved in regulation of the Hsp90 chaperone machinery.

## Introduction

The heat shock protein 90 (Hsp90) is a highly abundant and ubiquitous molecular chaperone that plays an essential role in the proper folding, stabilization and activation of a myriad of "client” proteins involved in various cellular processes. Hsp90 is important for stress response and seems to be a key player in maintaining cellular homeostasis [1,2]. Since Hsp90 and its client proteins are involved in multiple oncogenic signal transduction pathways crucial for growth and survival of cancer cells [3–6], it is important to understand the complexity of Hsp90 regulation. Human cytosolic Hsp90 (Hsp90α and Hsp90β) contains the amino-terminal (N-terminal) ATP-binding domain, the middle (M) domain and the carboxy-terminal (C-terminal) dimerization domain [1]. Hsp90 activity depends on ATP hydrolysis coupled with cyclic conformational rearrangements and is tightly regulated by sequential binding and release of different co-chaperones as well as by post-translational modifications [7–11]. Various co-chaperones can influence Hsp90 activity in different ways, including regulation of ATP hydrolysis and conformational dynamics or recruitment of specific client proteins. Among Hsp90 co-chaperones is Sgt1 (Suppressor of G2 allele of Skp1), originally discovered in yeast [12] and then in human cells where it is expressed in two isoforms, Sgt1A and Sgt1B. Sgt1 is composed of three domains: the N-terminal TPR (tetratricopeptide repeat) and middle CS (CHORD-containing proteins and Sgt1) domains, which are common to other co-chaperones, and the C-terminal SGS (Sgt1-specific) domain. It has been shown that Sgt1 binds Hsp90 through its CS domain [13] but further studies have revealed that also the SGS domain is required for the Sgt1-Hsp90 interaction [14]. Interestingly, the amino acid sequence of both CS and SGS domains of Sgt1 is highly similar to the sequence of the corresponding domains present in the CacyBP/SIP protein [13].

CacyBP/SIP [calcyclin (S100A6)-binding protein and Siah-1 interacting protein] was originally identified in Ehrlich ascites tumor cells as an S100A6 binding partner [15,16] and later described as a Siah-1 ligand [17]. CacyBP/SIP is present in various mammalian cells and tissues and seems to be involved in protein dephosphorylation, ubiquitination, cytoskeletal dynamics, regulation of gene expression, cell proliferation, differentiation and tumorigenesis [18]. Involvement of CacyBP/SIP in these processes might be due to the interaction with many targets such as members of the S100 family, Skp1 and Siah-1, cytoskeletal proteins and ERK1/2 kinase [18,19]. Recently, it has been reported that CacyBP/SIP shares sequence similarity with the MKP family phosphatases and is able to dephosphorylate ERK1/2 [20]. Moreover, CacyBP/SIP exhibits phosphatase activity towards tau protein, which suggests a possible role of CacyBP/SIP in Alzheimer’s disease pathology [21].

On the basis of the homology between Sgt1 and CacyBP/SIP and some preliminary data [13,22,23] we hypothesized that CacyBP/SIP might directly interact with Hsp90 and exhibit co-chaperone or chaperone properties. To confirm that, in this work we performed a set of experiments either using cell lysates or recombinant proteins. Moreover, taking into account that CacyBP/SIP exhibits phosphatase activity towards ERK1/2 we examined whether CacyBP/SIP might dephosphorylate Hsp90.

## Materials and Methods

### Cell culture, radicicol/novobiocin treatment and cell lysate preparation

HEp-2 (Human Epidermal Carcinoma) cells (ATCC) were cultured in DMEM (Sigma-Aldrich) supplemented with 10% (v/v) fetal bovine serum (FBS, Gibco), 40 mM sodium bicarbonate (Sigma-Aldrich), penicillin (100 units/ml) and streptomycin (100 μg/ml; both from Sigma-Aldrich) in 5% CO_2_ incubator at 37°C. The medium was changed every 2–3 days and the cells were passaged when confluent.

In order to examine the influence of Hsp90 inhibition on the CacyBP/SIP protein level, 70–80% confluent HEp-2 cells were incubated for 6 hrs with increasing concentrations of two different inhibitors of Hsp90 i.e., radicicol (5, 10 or 20 μM) and novobiocin (1, 2.5 or 5 mM) (Sigma-Aldrich). After that, total cell lysates were prepared in lysis buffer containing 25 mM Tris-HCl pH, 7.5, 150 mM NaCl, 1 mM EDTA, 1 mM EGTA, 0.5% (v/v) IGEPAL, 1% (v/v) Triton X-100, supplemented with protease inhibitors (Roche). The lysates were centrifuged at 20 000 x g for 20 min at 4°C and protein concentration was measured by the Bradford’s procedure [24]. Next, 30 μg of total protein from the supernatant was precipitated with cold acetone and analyzed by Western blot using specific anti-CacyBP/SIP antibody (Cell Signaling Technology).

### Cell transfection and co-immunoprecipitation

In order to check whether CacyBP/SIP co-precipitates with Hsp90, HEp-2 cells were transfected with an appropriate plasmid: pcDNA3.1-3xFLAG-Hsp90α, pcDNA3.1-3xFLAG-Hsp90β, p3xFLAG-CMV-10-Hsp90β(N+M) [encoding fragment covering the N-terminal (N) and middle (M) domain, residues 1–546], p3xFLAG-CMV-10-Hsp90β(M+C) [encoding fragment covering the middle (M) and C-terminal (C) domain, residues 206–724], p3xFLAG-CMV-10-Hsp90β(M) [encoding fragment covering the middle (M) domain, residues 206–546] or with a control plasmid p3xFLAG-CMV-10, using TrueFect Transfection Reagent (United Biosystems) according to the manufacturer's protocol. After 5 hrs, the medium was replaced with fresh complete medium and cells were cultured in 5% CO_2_ at 37°C for the next 24 hrs. For co-immunoprecipitation (co-IP) cells were washed with ice-cold PBS, harvested and homogenized mechanically by being passed 40 times through a needle (0.45 x 13 mm) in the IP buffer containing 10 mM Tris-HCl, pH 7.5, 50 mM KCl, 3 mM MgCl_2_, 0.1% (v/v) Triton X-100 supplemented with protease and phosphatase inhibitors (Roche). Cell lysate was centrifuged at 16 000 x g for 10 min at 4°C and protein concentration was measured by the Bradford’s procedure. Next, approximately 800–1000 μg of total protein from the supernatant fraction was incubated with anti-FLAG antibody coupled to agarose beads (Sigma-Aldrich) at 4°C for 3 hrs. The unbound fraction was removed, the resin was washed with IP buffer and the bound proteins were eluted from the anti-FLAG resin using 0.1 M glycine, pH 3.5. The eluted proteins were precipitated with cold acetone and analyzed by Western blot using specific antibodies. In order to examine the influence of Hsp90 inhibition on the CacyBP/SIP-Hsp90 complex formation, cells were treated with 1 μM radicicol or 1 mM novobiocin for 2 hrs and then harvested as described above.

### SDS-PAGE and immunoblotting

Gel electrophoresis with 10% (v/v) polyacrylamide was performed by the method of Laemmli [25]. Then, the proteins were transferred electrophoretically onto nitrocellulose membrane (BioRad) and identified using appropriate primary antibodies: polyclonal rabbit anti-CacyBP/SIP (Cell Signaling Technology; dilution 1:1000), mouse monoclonal anti-Sgt1 and mouse monoclonal anti-Hsp90 (BD Transduction Laboratories; dilution 1:500 and 1:1000, respectively), rat monoclonal anti-Hsp90 (Enzo Life Sciences; dilution 1:1000), rabbit polyclonal anti-Raf1 (Santa Cruz Biotechnologies; dilution 1:1000), mouse monoclonal anti-FLAG (Sigma-Aldrich; dilution 1:2500) and mouse monoclonal anti-β-actin HRP-conjugated (Sigma-Aldrich, dilution 1:40000). Incubation with primary antibodies was carried out overnight at 4°C. After washing with TBS-T containing 50 mM Tris-HCl, pH 7.5, 200 mM NaCl and 0.05% (v/v) Tween-20, the membranes were allowed to react with appropriate secondary antibodies conjugated to horseradish peroxidase (HRP): goat anti-mouse IgG (Jackson Immunoresearch Laboratories, dilution 1:12000), goat anti-rabbit IgG (MP Biomedicals, dilution 1:10000) or rabbit anti-rat IgG (Enzo Life Sciences; dilution 1:5000). The memmbranes were then washed with TBS-T and developed using Clarity Western ECL Substrate (BioRad) followed by exposure against an X-ray film. The intensities of protein bands were quantified, with β-actin as a reference protein, using the Ingenius densitometer and the Gene Tools software (SynGene). Statistical data analysis was performed using the Student's *t*-test.

### Two-dimensional (2D) electrophoresis

HEp-2 cells were co-transfected with plasmid encoding Hsp90β-3xFLAG and either CacyBP/SIP-3xFLAG or 3xFLAG (control), analogically as described above. 24 hrs after transfection, total cell lysates were prepared in lysis buffer described above, supplemented with protease inhibitors (Roche). Lysates prepared from cells co-transfected with control plasmid, p3xFLAG-CMV-10, were incubated or not with Lambda Protein Phosphatase (Lambda PP; New England BioLabs). Then, 250 μg of protein precipitated using ReadyPrep 2D Cleanup kit (BioRad) was resuspended in sample buffer containing 8 M urea, 2% (w/v) CHAPS, 50 mM DTT and 0.2% (v/v) ampholyte (BioLyte pH 3–10; BioRad). Samples were applied on linear 7 cm long pH-gradient (pH 3–10) strips (BioRad) and incubated for 16 hrs at RT for rehydration. Following the rehydration, isoelectrofocusing (IEF) was performed for 16 hrs with final voltage of 4000 V using the Protean IEF Cell instrument (BioRad). Next, the strips were equilibrated for 25 min in the buffer containing 375 mM Tris-HCl, pH 8.8, 6 M urea, 2% (w/v) SDS, 20% (v/v) glycerol and 2% (w/v) DTT and then for 25 min in the buffer containing 2% (w/v) iodoacetamide instead of DTT. Finally, standard SDS-PAGE with 10% (v/v) polyacrylamide gel was performed followed by Western blot analysis using mouse monoclonal anti-Hsp90 antibody (BD Transduction Laboratories).

In another approach, 2 μg of Hsp90 purified from HeLa cells (Enzo Life Sciences) was incubated alone (control) or in the presence of 4 μg of recombinant CacyBP/SIP in a buffer containing 20 mM Tris-HCl, pH 8.4, 150 mM NaCl for 30 min at 30°C. For positive control, Hsp90 was incubated with 0.5 μl (20 U) of Lambda Protein Phosphatase (Lambda PP; New England BioLabs), instead of CacyBP/SIP, in a buffer containing 20 mM Tris-HCl, pH 7.5, 150 mM NaCl and 1 mM MnCl_2_. Then, the proteins were precipitated using the ReadyPrep 2D Cleanup kit (BioRad) and analyzed by 2D electrophoresis and Western blot as described above.

### Proximity ligation assay

To visualize the CacyBP/SIP-Hsp90 complexes (and Sgt1-Hsp90 ones as a positive control) in HEp-2 cells, the proximity ligation assay (PLA; In situ PLA Technology, Olink Bioscience) was applied. Cells grown on poly-L-lysine (Sigma-Aldrich) coated coverslips, were fixed with 3% (w/v) paraformaldehyde in the PHEM buffer containing 60 mM PIPES, 25 mM HEPES, pH 6.9, 5 mM EGTA, 4 mM MgCl_2_ for 20 min at RT.

The coverslips were then washed with PBS, incubated with 50 mM NH_4_Cl in the PHEM buffer for 10 min at RT and washed with PBS. Next, cells were permeabilized for 4 min with 0.1% (v/v) Triton X-100 in the PHEM buffer and washed again with PBS. All the following steps were performed according to the manufacturer's protocol using reagents (except primary antibodies) and buffers provided in the PLA kit. The reaction with primary antibodies, mouse anti-Hsp90 (BD Transduction Laboratories) and either rabbit anti-CacyBP/SIP (Cell Signaling Technology) or rabbit anti-Sgt1 (ProteinTech) (each diluted 1:100) was conducted for 2 hrs at RT in a humidity chamber. In control experiment, cells were incubated under the same conditions without primary antibodies or the ligase or with primary antibodies against ATP synthase C (a protein not interacting with Hsp90). After washing, the incubation with anti-rabbit PLUS and anti-mouse MINUS PLA-probes (1:5) was carried out for 1 hr at 37°C in a humidity chamber. Following the ligation and amplification steps, the coverslips were immobilized on the glass slides with the mounting medium containing DAPI. The cells were analyzed with Zeiss Spinning Disc Confocal Microscope (Carl Zeiss GmbH) with 63×oil objective in the Laboratory of Imaging Tissue Structure and Function (Nencki Institute of Experimental Biology PAS).

### Plasmids

For cell transfection the following plasmids were used: pcDNA3.1(+)-3xFLAG-HA-Hsp90α, pcDNA3.1(+)-3xFLAG-HA-Hsp90β, p3xFLAG-CMV-10-Hsp90β(N+M), p3xFLAG-CMV-10-Hsp90β(M+C), p3xFLAG-CMV-10-Hsp90β(M), and p3xFLAG-CMV-10-CacyBP/SIP. Construction of the pcDNA3.1(+) plasmids encoding full length human Hsp90α and Hsp90β with N-terminal FLAG and HA tags was described by Zurawska *et al*., [26]. The p3xFLAG-CMV-10-Hsp90β(N+M), p3xFLAG-CMV-10-Hsp90β(M+C) and p3xFLAG-CMV-10-Hsp90β(M) plasmids encoding the N-terminal (N) and the middle (M) domains (residues 1–546), the middle (M) and the C-terminal (C) domains (residues 206–724) or the middle (M) domain (residues 206–546) of Hsp90β, respectively, were obtained by PCR using appropriate primers and the p423TDH3-Hsp90β plasmid as a template [26]. The plasmid encoding human CacyBP/SIP with the N-terminal FLAG tag was prepared as follows. cDNA obtained from total RNA purified from SH-SY5Y cells served as a template for PCR reaction with appropriate primers (Table 1) to amplify human CacyBP/SIP sequence which was then cloned into p3xFLAG-CMV-10 plasmid.

**Table 1:** List of restriction enzymes and primers used for cloning.

Plasmid	Restriction site	Sequence of primer/orientation
pFLAG-Hsp90β-NM	EcoRV	F GATCTGATATCGCCTGAGGAAGTGCACC
pFLAG-Hsp90β-NM	BglII	R GGTCCAGATCTTCAATCCTCAGGCAGCTCCAG
pFLAG-Hsp90β-MC	EcoRV	F GATCTGATATCTTCTCAGTTCATAGGCTATCC
pFLAG-Hsp90β-MC	BglII	R GCAGCAGATCTCTAATCGACTTCTTCCATGC
pFLAG-Hsp90β-M	EcoRV	F GATCTGATATCTTCTCAGTTCATAGGCTATCC
pFLAG-Hsp90β-M	BglII	R GGTCCAGATCTTCAATCCTCAGGCAGCTCCAG
pFLAG-CacyBP/SIP	HindIII	F TATAAAGCTTATGGCTTCAGAAGAGCTACA
pFLAG-CacyBP/SIP	BamHI	R TATAGGATCCTCAAAATTCCGTGTCTCCTT
His6-Sgt1B	NdeI	F ATAATTCATATGGCGGCGGCTGCAGCA
His6-Sgt1B	BamHI	R ACGCGCGGATCCTTAGTACTTTTTCCATTC

Plasmids were constructed in pCMV10-FLAG and pET28b using restriction enzymes incorporated in the sequence of the primers (underlined). Orientation of the primers is indicated as F (forward) and R (reverse).

For synthesis and purification of recombinant proteins, pET28a(+)-CacyBP/SIP-His, pET28a(+)-Sgt1A-His and pET28a(+)-Sgt1B-His plasmids were used. Construction of the pET28a(+) plasmids encoding mouse CacyBP/SIP and human Sgt1A with N-terminal His tag were described by Filipek *et al*., [27] and Nowotny *et al*., [28], respectively. To generate the pET28a(+)-Sgt1B-His plasmid, appropriate primers (Table 1) and the pDEST17-hSgt1B plasmid template (generously provided by Prof. Katsumi Kitagawa, University of British Columbia, Vancouver, Canada) were used. The amplified fragment encoding Sgt1B was then cloned into the pET28a(+) plasmid (Novagen). Sequences of all primers and restriction enzymes used for cloning are listed in Table 1.

### Expression and purification of recombinant proteins

All His-tagged proteins were expressed in *E*. *coli* (BL21 DE3 strain) as described previously [28] and purified at RT by metal affinity chromatography using Ni^2+^-NTA (for Sgt1A and Sgt1B) or TALON Co^2+^ (for CacyBP/SIP) resins (Clontech). The His-tag was removed by thrombin (Novagen) cleavage according to the manufacturer's instruction. Proteins were dialyzed against 25 mM Tris-HCl, pH 8.0, 50 mM NaCl (Sgt1A and Sgt1B) or 20 mM Tris-HCl, pH 8.4, 150 mM NaCl (CacyBP/SIP) and concentrated using centrifugal filters (Amicon, Millipore).

Size-exclusion chromatography (SEC) in fast protein liquid chromatography (FPLC) system was applied as the last preparative step of protein purification. SEC was performed using an ÄKTA Purifier machine (GE Healthcare Life Sciences) and HiLoad™ 16/600 Superdex™ 200 analytical gel filtration column (total resin volume of 120 ml). Data were analyzed using the UNICORN 5.31 software. The column was equilibrated with the same buffer as used for dialysis and then protein sample in this buffer was loaded with flow rate 1 ml/min and pressure of approximately 0.45 MPa. In order to separate the protein sample from non-specific aggregates, only fractions from the main peak were collected, concentrated and, if necessary, enriched with glycerol up to 10% (v/v) for storage.

### ELISA assays

Hsp90 purified from HeLa cells (Enzo Life Sciences) and BSA (Serva) were coated onto 96-well microtiter plates (500 ng/well) in 50 μl of Coating Buffer (CB) containing 25 mM HEPES, pH 7.6 and 100 mM KCl. After overnight incubation at 4°C, the solution was removed and the wells were washed with Washing Buffer (WB) containing 25 mM HEPES, pH 7.6, 100 mM KCl and 2% (w/v) BSA. The remaining absorption sites were blocked for 3 hrs at RT with WB. Then, wells were washed with Reaction Buffer (RB) containing 25 mM HEPES, pH 7.6, 50 mM KCl, 5 mM MgCl_2_, 5% (v/v) glycerol, 2 mg/ml BSA, 2 mM DTT and 0.05% (v/v) Triton X-100 and increasing concentrations (0 to 1.33 μM) of recombinant CacyBP/SIP were applied in 50 μl of RB. In the competition assay 0.67 μM concentration of recombinant Sgt1 (Sgt1A and Sgt1B in a molar ratio 1:1) was added together with CacyBP/SIP or, in the second variant of the experiment, constant concentration (1.33 μM) of CacyBP/SIP was added alone or with increasing concentrations (0 to 1.33 μM) of Sgt1 (Sgt1A and Sgt1B in a molar ratio 1:1). After overnight incubation at 4°C, the wells were washed with PBS supplemented with 0.05% (v/v) Tween-20 (PBS-T) and the primary rabbit polyclonal antibody against CacyBP/SIP (Cell Signaling Technology) diluted 1:250 in WB was applied for 3 hrs at RT. Following the washing procedure with PBS-T, incubation with secondary HRP-conjugated anti-rabbit antibody (MP Biomedicals) diluted 1:6000 in WB was carried for 2 hrs at RT. After the final wash with PBS-T, the bound antibodies were detected calorimetrically using the TMB peroxidase EIA substrate (Millipore) according to the manufacturer's instructions. The reaction was stopped with 1 M sulfuric acid and the absorbance was measured at 450 nm using a microplate reader (ThermoLabsystem).

### Luciferase renaturation assay

Recombinant firefly luciferase (Promega) was diluted in buffer containing 25 mM HEPES, pH 7.4, 50 mM KCl, 5 mM MgCl_2_ and 1 mM ATP, to 0.04 μM concentration and then thermally denatured in 31°C for 3 min in the absence or presence of 0.5 μM Hsp90, 0.8 μM CacyBP/SIP or both. Next, after 1 min incubation on ice, the renaturation reaction was allowed to proceed up to 120 min at 25°C. For luciferase activity measurement with a luminometer (Turner Designs), aliquots of 1 μl were taken from the renaturation reactions and mixed with 20 μl of luciferase assay reagent (Promega). In the second variant of the experiment other recombinant chaperones, 0.8 μM Hsp40 and 0.4 μM Hsp70 (Enzo Life Sciences), were added to each sample directly after denaturation and renaturation was allowed to proceed for up to 90 min at 25°C.

### Citrate synthase aggregation assay

Porcine citrate synthase (Sigma Aldrich) was assayed for heat-induced aggregation as described previously [29], citrate synthase (CS), prepared in a buffer containing 40 mM HEPES, pH 7.5 and 1 mM ATP, was incubated at 43°C for 21 min alone (control) or in the presence of recombinant CacyBP/SIP, Hsp90 purified from HeLa cells (Enzo Life Sciences) or both proteins. As a negative control CS was incubated with recombinant GST (glutathione S-transferase, a gift from Ewelina Jurewicz, Nencki Institute of Experimental Biology, Warsaw). All proteins were used at 0.15 μM concentration. To determine the extent of CS aggregation, the increase in optical density due to light scattering was measured at 360 nm using thermostated spectrofluorimeter (Hitachi F-7000 Fluorescence Spectrophotometer) and the FL Solutions F-7000 software.

## Results

### CacyBP/SIP interacts with Hsp90 in the cell and under *in vitro* conditions

**Fig 1:**
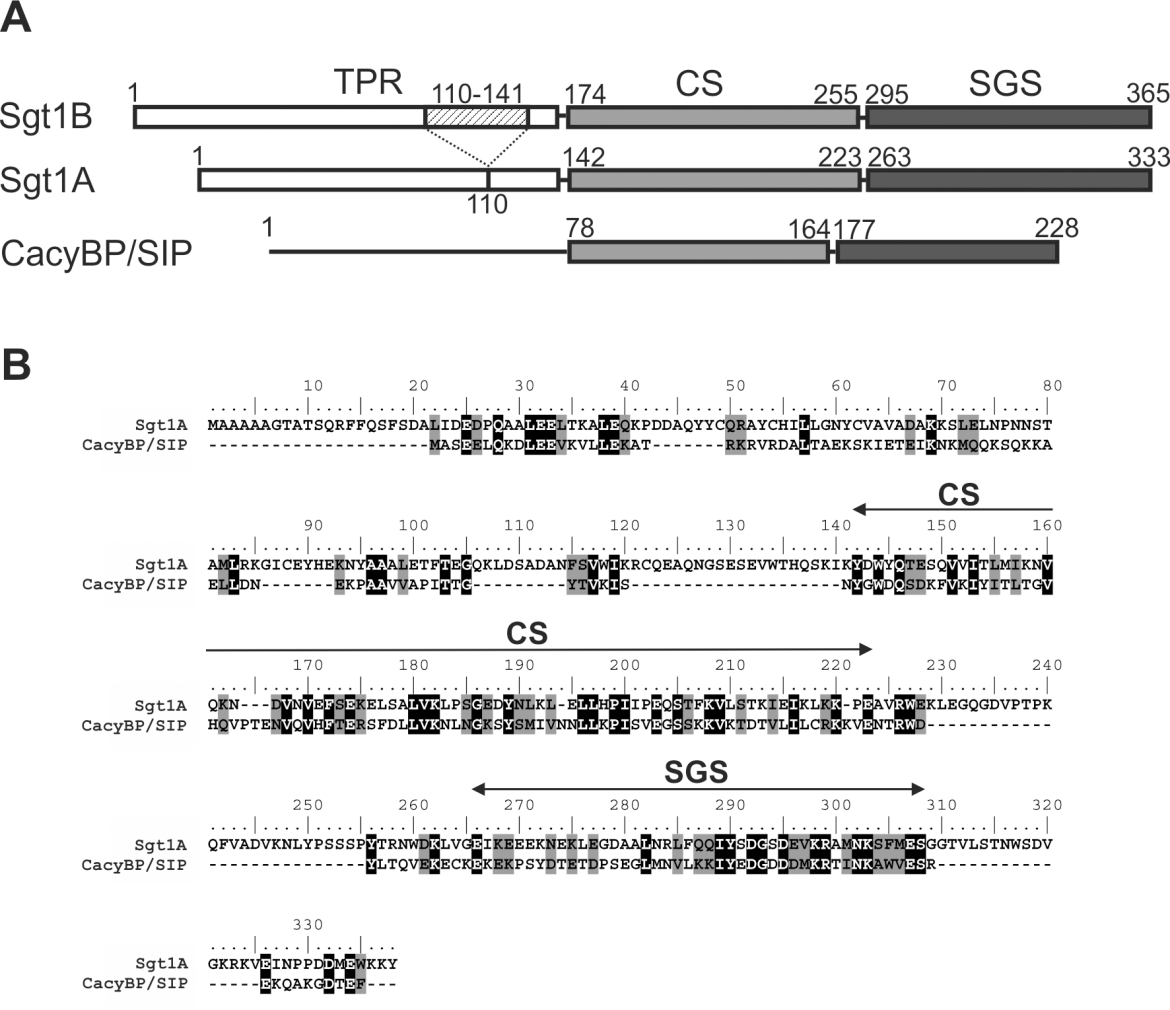
Comparison of human Sgt1 and CacyBP/SIP. **(A)** Schematic representation of CacyBP/SIP and both isoforms of Sgt1 (Sgt1A and Sgt1B). Grey and black boxes show the CS (CHORD-containing proteins and Sgt1) and the SGS (Sgt1-specific) domains. Hatched box indicates the fragment of sequence specific for Sgt1B. **(B)** Sequence alignment of CacyBP/SIP and Sgt1A. The sequences were compared using ClustalW software. Black and grey boxes indicate identical and similar amino acid residues, respectively. The amino acid identity and similarity in the SGS domain are 29.3% and 63.4%, respectively, and in the CS domain are 36.8% and 55.2%, respectively.

Since the amino acid sequence of the CS and SGS domains of Sgt1, a known Hsp90 co-chaperone, is highly similar to the sequence of the corresponding domains present in the CacyBP/SIP protein (Fig 1) we checked if CacyBP/SIP might directly interact with Hsp90 and exhibit co-chaperone or chaperone properties.

**Fig 2:**
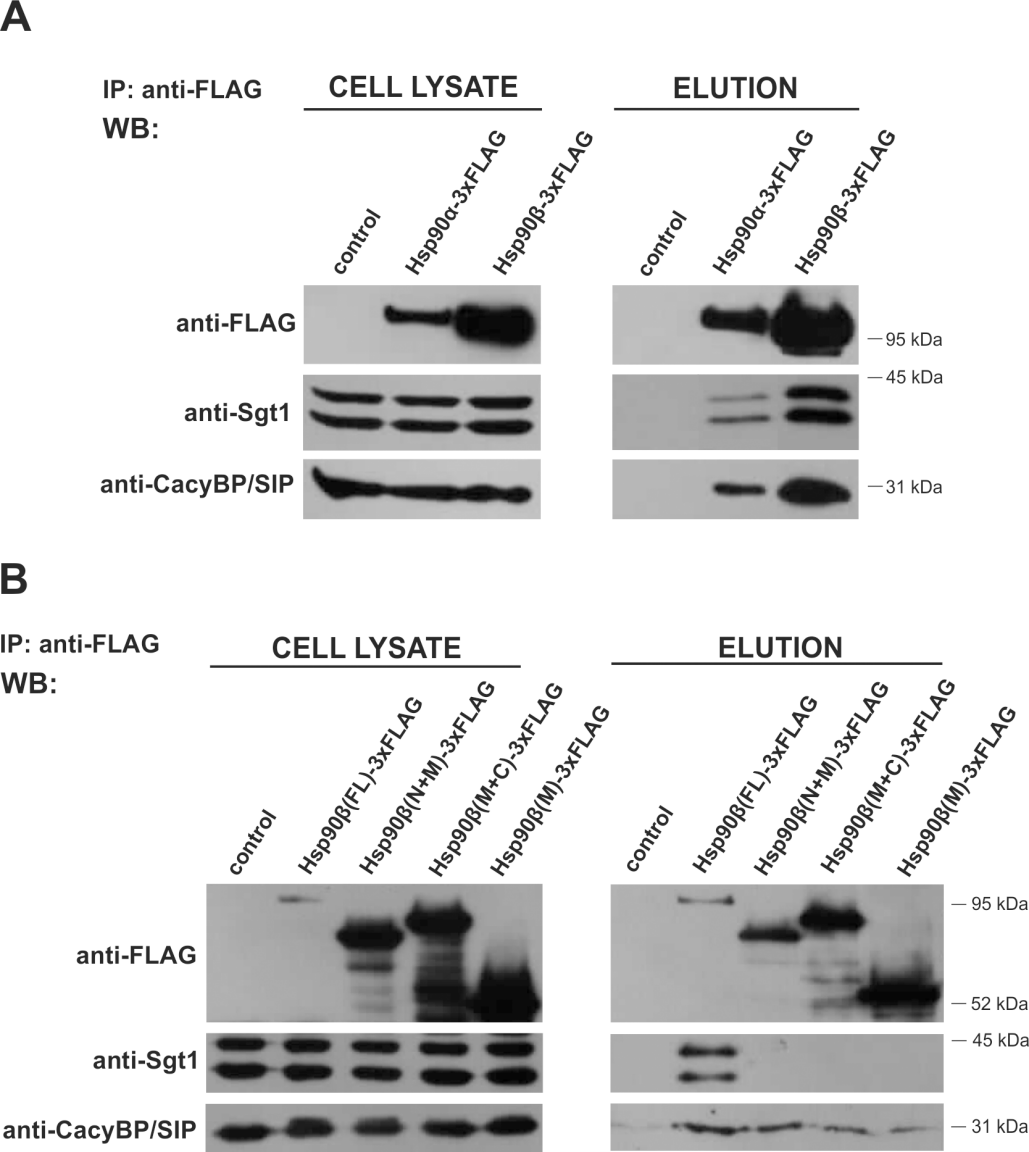
Hsp90-CacyBP/SIP complex formation. Co-immunoprecipitation of CacyBP/SIP and Sgt1 with **(A)** FLAG-tagged Hsp90α and Hsp90β or **(B)** full-length Hsp90β (FL) and its deletion mutants containing N-terminal + middle domains (N+M), middle + C-terminal (M+C) or middle (M) domains. Anti-FLAG staining shows expression of Hsp90α-3xFLAG, Hsp90β-3xFLAG and FLAG-tagged Hsp90β deletion mutants. For control, HEp-2 cells were transfected with plasmid encoding 3xFLAG alone. A representative Western blot, out of 3 performed, is shown.

At first, in order to establish whether CacyBP/SIP binds to Hsp90 we expressed Hsp90α-3xFLAG or Hsp90β-3xFLAG in HEp-2 cells and then performed co-immunoprecipitation using anti-FLAG antibody coupled to agarose beads. As it is shown in Fig 2A, not only Sgt1 but also CacyBP/SIP are bound to both FLAG-tagged Hsp90 isoforms. To check which domain of Hsp90 is involved in the interaction with CacyBP/SIP, we performed co-immunoprecipitation on anti-FLAG agarose using lysates of HEp-2 cells with expression of FLAG-tagged full length Hsp90β(FL) or its three deletion mutants: Hsp90β(N+M), Hsp90β(M+C) and Hsp90β(M). Western blot analysis of proteins eluted from the resin indicates that CacyBP/SIP, in contrast to Sgt1, co-precipitates not only with full length Hsp90β(FL)-3xFLAG but also with its deletion mutants: Hsp90β(N+M)-3xFLAG, Hsp90β(M+C)-3xFLAG and Hsp90(M)-3xFLAG (Fig 2B).

**Fig 3:**
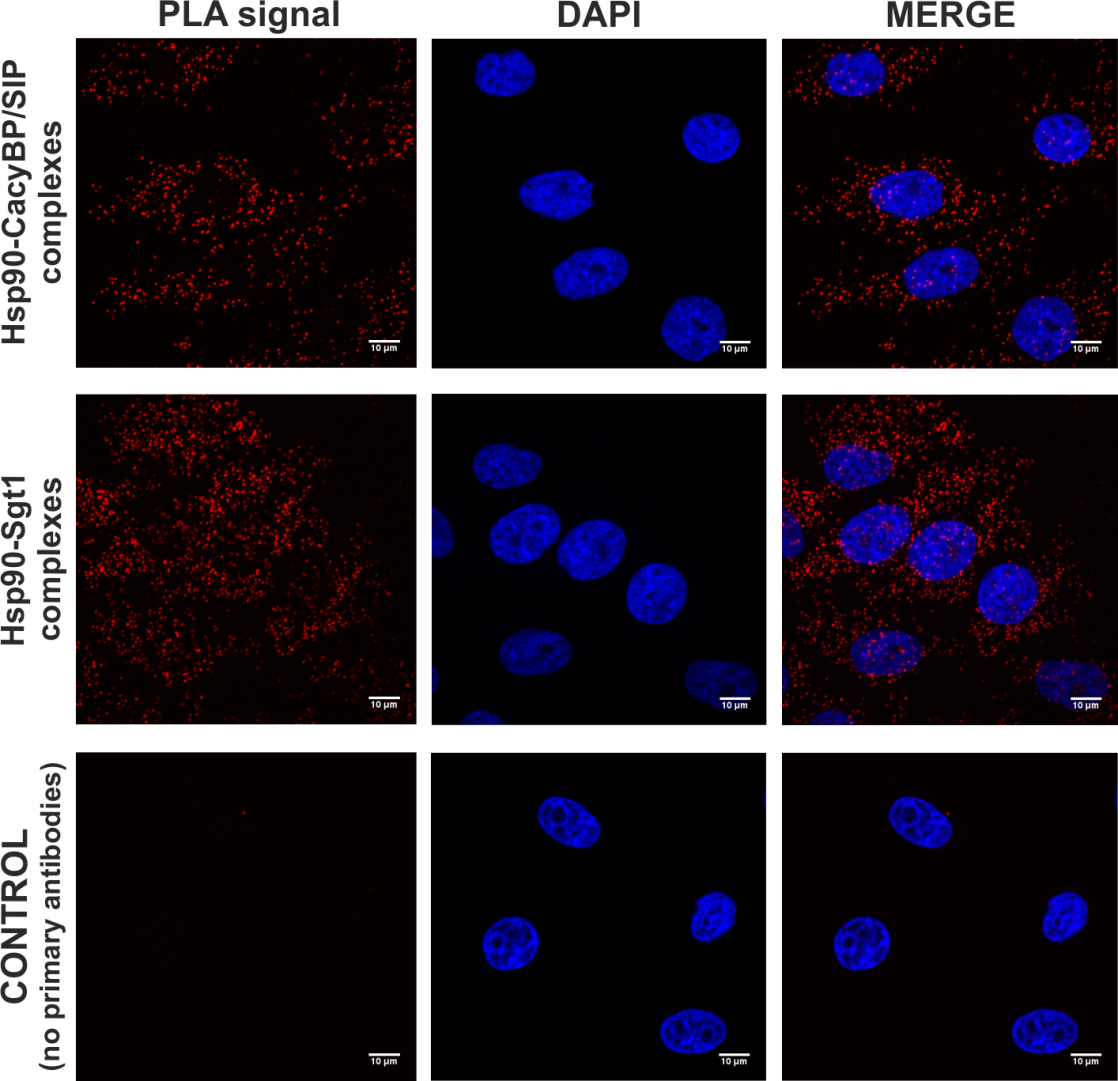
Presence of Hsp90-CacyBP/SIP and Hsp90-Sgt1 complexes in HEp-2 cells analyzed by PLA assay. Complexes of examined proteins are visualized in red; cell nuclei, stained with DAPI, are in blue. Scale bar is 10 μm.

Furthermore, the proximity ligation assay (PLA) performed on HEp-2 cells with the use of anti-Hsp90 and anti-CacyBP/SIP antibodies, showed that both CacyBP/SIP and Sgt1 form complexes with Hsp90 in the cytoplasm (Fig 3, upper and middle panel, respectively). In control experiment, cells were incubated without primary antibodies and no signals are detected (Fig 3, bottom panel). To prove that the interactions observed for Hsp90-Sgt1 and Hsp90-CacyBP/SIP are specific, three additional controls were performed. In the first one, antibodies against Hsp90 alone were included, in the second one, the ligase, a critical reagent in PLA assay, was omitted and in the third one, the primary antibodies against Hsp90 were included together with antibodies against ATP synthase C, a protein which does not bind to Hsp90. In all cases no signals were detected (S1 Fig).

**Fig 4:**
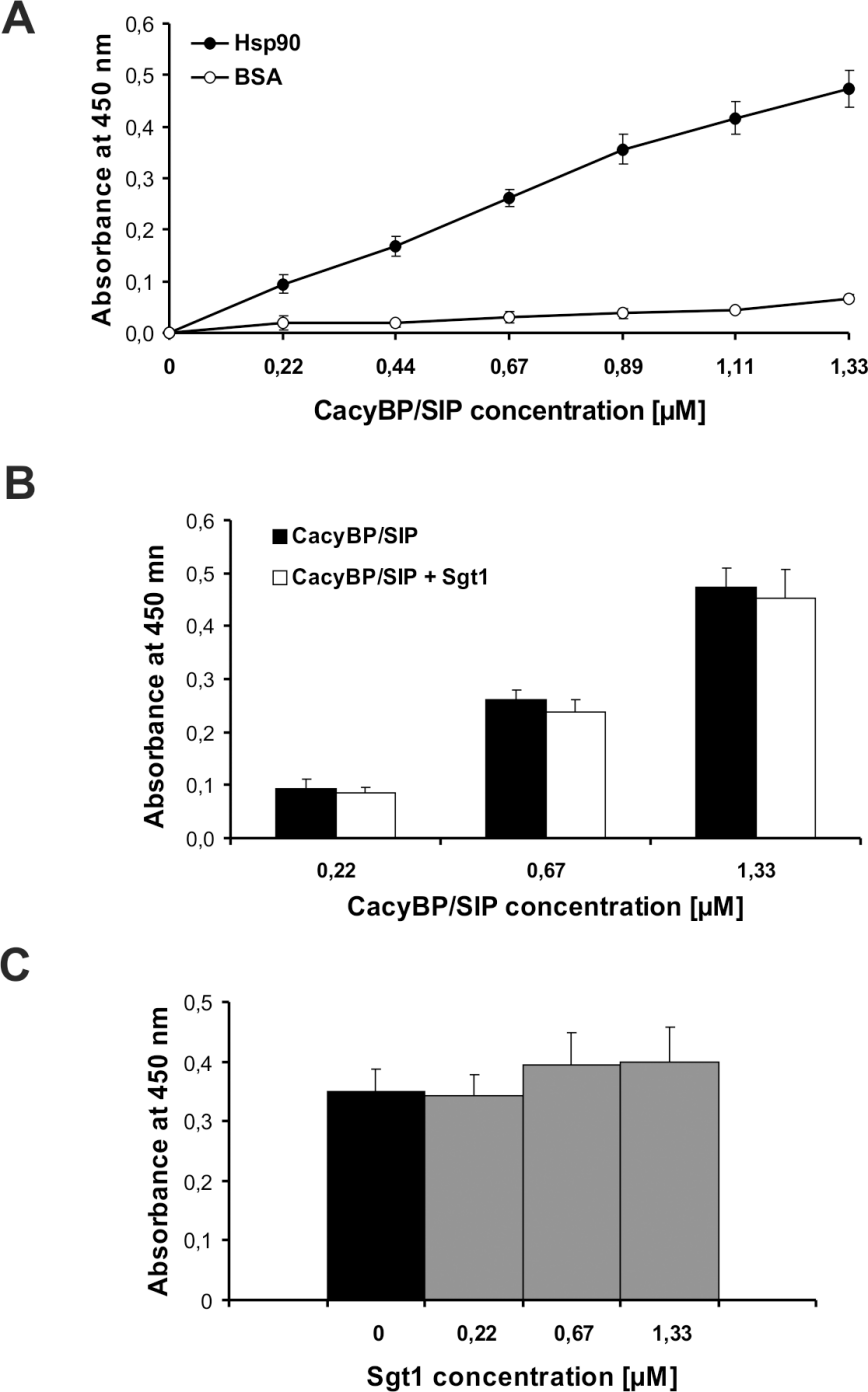
Binding of purified Hsp90 to CacyBP/SIP. (**A**) ELISA assay with increasing concentrations of CacyBP/SIP. **(B)** Competitive ELISA assay with increasing concentrations of CacyBP/SIP in the absence (black bars) or presence (white bars) of constant concentration of Sgt1 (0.67 μM). **(C)** Competitive ELISA assay with constant concentration of CacyBP/SIP (1.33 μM) in the absence (black bar) or in the presence of increasing concentrations of Sgt1 (grey bars). The results from 3 independent experiments are presented as a mean value of absorbance ± standard error (SEM).

Next, to examine whether Hsp90 and CacyBP/SIP interact directly under *in vitro* conditions, ELISA assay with purified proteins was performed. Hsp90 was immobilized on 96-well microtiter plates and then incubated with increasing concentrations of CacyBP/SIP. Bound CacyBP/SIP was allowed to react with specific anti-CacyBP/SIP antibody and visualized by colorimetric detection. As it can be seen in Fig 4A, Hsp90 interacts with the CacyBP/SIP protein. However, lack of the signs of saturation even at the highest concentration of CacyBP/SIP suggests that the interaction might be weak and transient.

As it has been already mentioned, the CacyBP/SIP amino acid sequence is homologous to that of Sgt1. Thus, we determined whether both proteins have the same or different binding sites on the Hsp90 molecule. For that we performed competitive ELISA assay in which immobilized Hsp90 was incubated with increasing concentrations of CacyBP/SIP (molar ratio of CacyBP/SIP to Hsp90 ranged from 2:1 to 12:1) in the absence or in the presence of constant amount of Sgt1 (molar ratio of Sgt1 to Hsp90 was 6:1). We have found that there is no statistically significant difference between the signals from wells where CacyBP/SIP was added alone and the wells were CacyBP/SIP was present together with Sgt1 (Fig 4B). Also, we checked whether Sgt1 could influence the CacyBP/SIP-Hsp90 interaction. As it was expected, increasing concentrations of Sgt1 (molar ratio of Sgt1 to Hsp90 ranged from 2:1 to 12:1) added to a constant amount of CacyBP/SIP (molar ratio of CacyBP/SIP to Hsp90 was 12:1) do not influence its binding to Hsp90 (Fig 4C). These results demonstrate that CacyBP/SIP and Sgt1 are able to bind to Hsp90 independently and therefore interact with distinct sites on the Hsp90 molecule. Taken together, our data indicate that CacyBP/SIP directly binds to Hsp90 and does not compete for the binding with Sgt1, a known Hsp90 co-chaperone.

### Hsp90 inhibition does not influence CacyBP/SIP-Hsp90 binding

**Fig 5:**
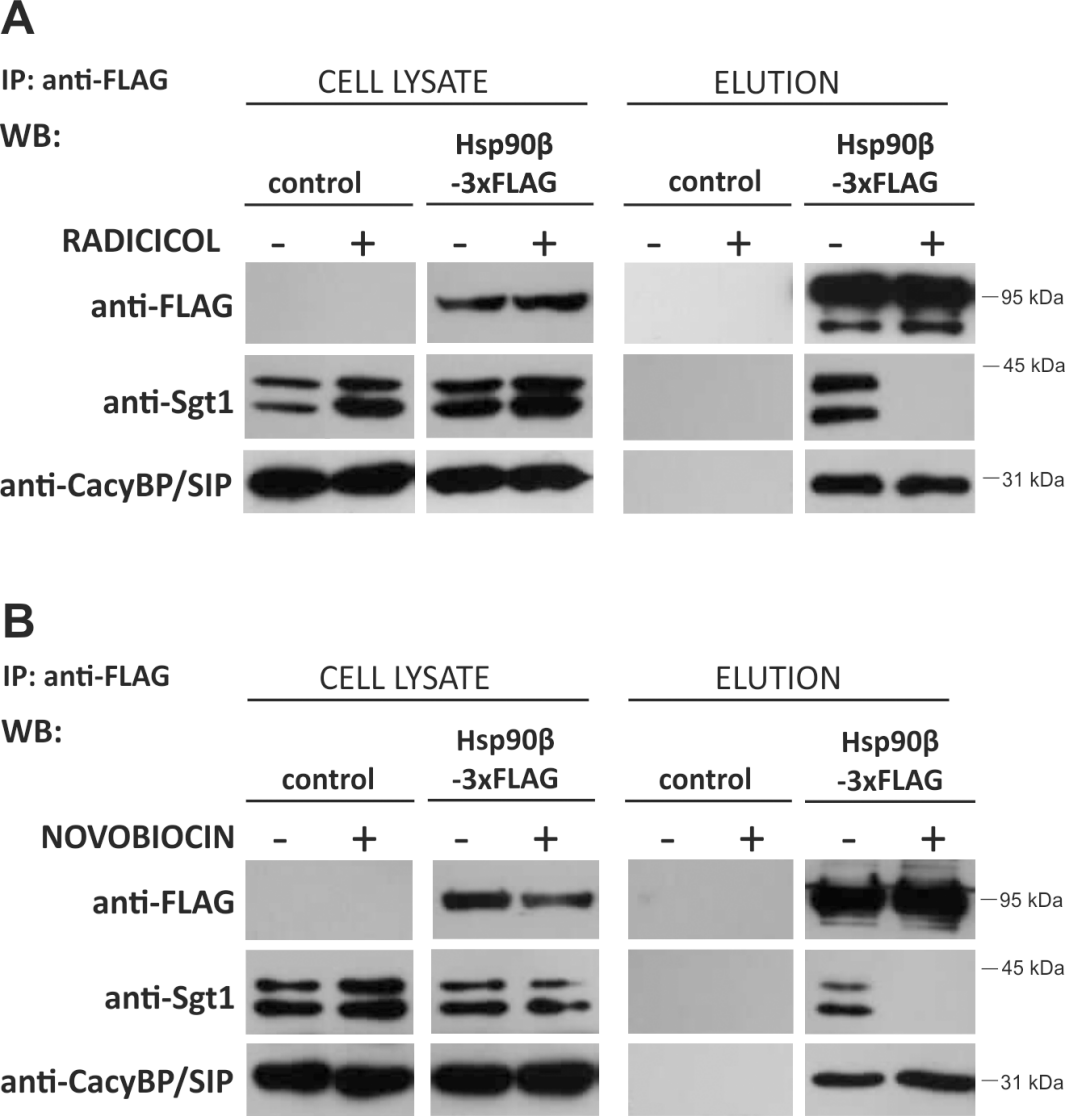
Influence of Hsp90 inhibition on Hsp90-CacyBP/SIP and Hsp90-Sgt1 complex formation. Co-immunoprecipitation of CacyBP/SIP and Sgt1 with FLAG-tagged Hsp90β from HEp-2 cell lysate obtained after 2 hrs treatment with radicicol **(A)** or novobiocin **(B)**. Anti-FLAG staining shows expression of FLAG-tagged Hsp90β. A representative Western blot, out of 3 performed, is shown.

**Fig 6:**
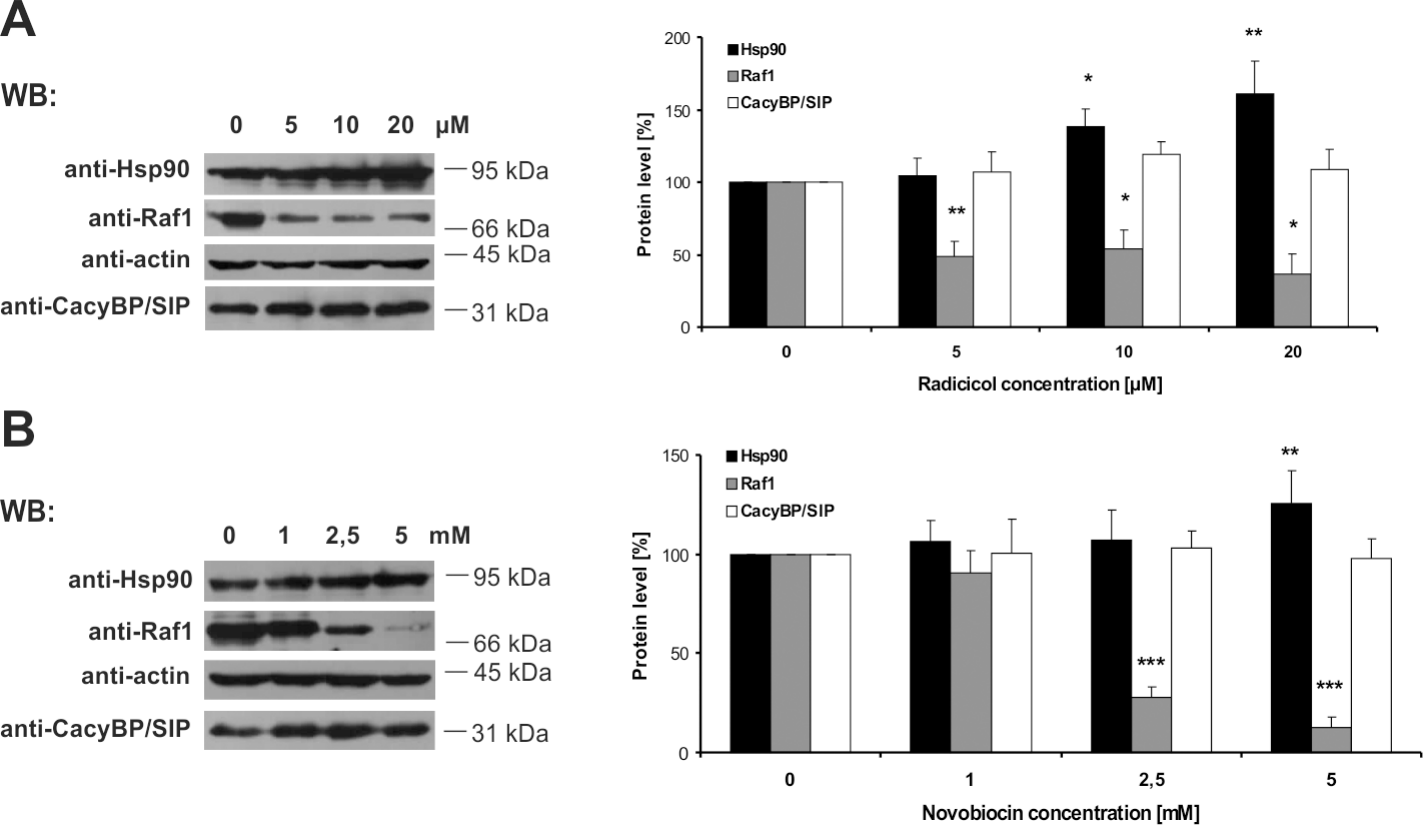
Influence of Hsp90 inhibition on CacyBP/SIP protein level. Level of CacyBP/SIP in HEp-2 cells treated with different concentrations of radicicol (**A**) or novobiocin (**B**). Representative Western blots (left panels) and densitometric analysis of data from 3 independent experiments (right panels) are shown as means ± standard errors (SEM); *** p ≤ 0.001, ** p ≤ 0.0.01, * p ≤ 0.05.

Next, we examined the influence of two Hsp90 inhibitors, radicicol and novobiocin, on CacyBP/SIP-Hsp90 complex formation and on CacyBP/SIP protein level in HEp-2 cells. Radicicol and novobiocin are naturally occurring antibiotics that bind to Hsp90 (N- and C-terminal domain, respectively) and inhibit Hsp90 activity which, in turn, might prevent the formation of Hsp90 chaperone complexes. Therefore, in order to check whether the activity of Hsp90 is prerequisite for CacyBP/SIP interaction, an immunoprecipitation assay using cell lysate obtained from HEp-2 cells transfected with plasmids encoding Hsp90β-3xFLAG was performed. As it can be seen in Fig 5, neither radicicol (Fig 5A) nor novobiocin (Fig 5B) abolishes the CacyBP/SIP-Hsp90 interaction. In contrast, Sgt1 co-immunoprecipitates only with active Hsp90 (without any inhibitor). To check whether the level of CacyBP/SIP changes in response to Hsp90 inactivation, HEp-2 cells were incubated with increasing concentrations of inhibitors for 6 hrs and then protein lysates were examined by Western blot. Densitometric analysis of the results, showed that the CacyBP/SIP protein level does not change upon treatment with increasing concentration of radicicol or novobiocin (Fig 6A and 6B). On the contrary, the level of the Raf-1 kinase, a Hsp90 client, decreases and the level of Hsp90 increases. Based on these results it can be concluded that CacyBP/SIP is not a Hsp90 client.

### CacyBP/SIP exhibits chaperone properties under *in vitro* conditions

**Fig 7:**
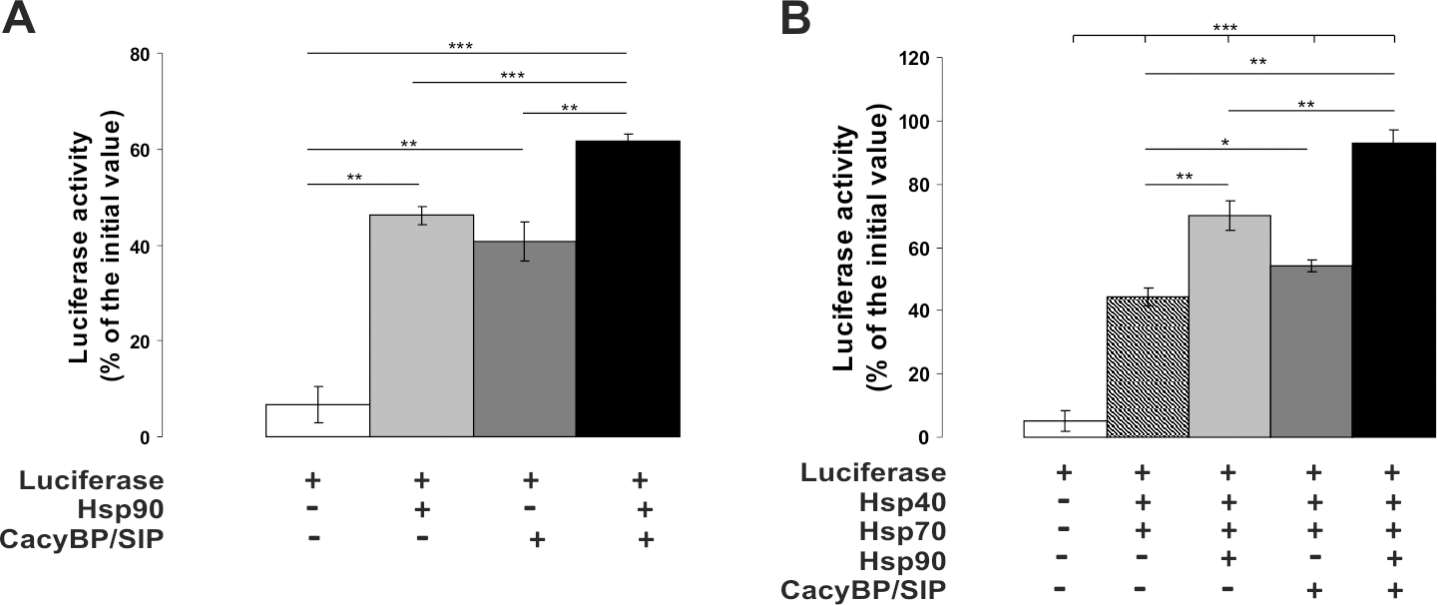
Renaturation of thermally denatured luciferase by CacyBP/SIP. Purified, recombinant luciferase (0.04 μM) was denatured alone (control) or in the presence of 0.5 μM Hsp90, 0.8 μM CacyBP/SIP or both at 31°C for 3 min and then left to recover at 25°C for 120 min **(A)** or for 90 min **(B)**. In **(B)** 0.8 μM Hsp40 and 0.4 μM Hsp70 were added directly after denaturation. Luciferase activity was calculated as a percentage of the value before denaturation. Data from 4 independent experiments are shown as a mean ± standard error (SEM); *** p ≤ 0.001, ** p ≤ 0.0.01, * p ≤ 0.05.

**Fig 8:**
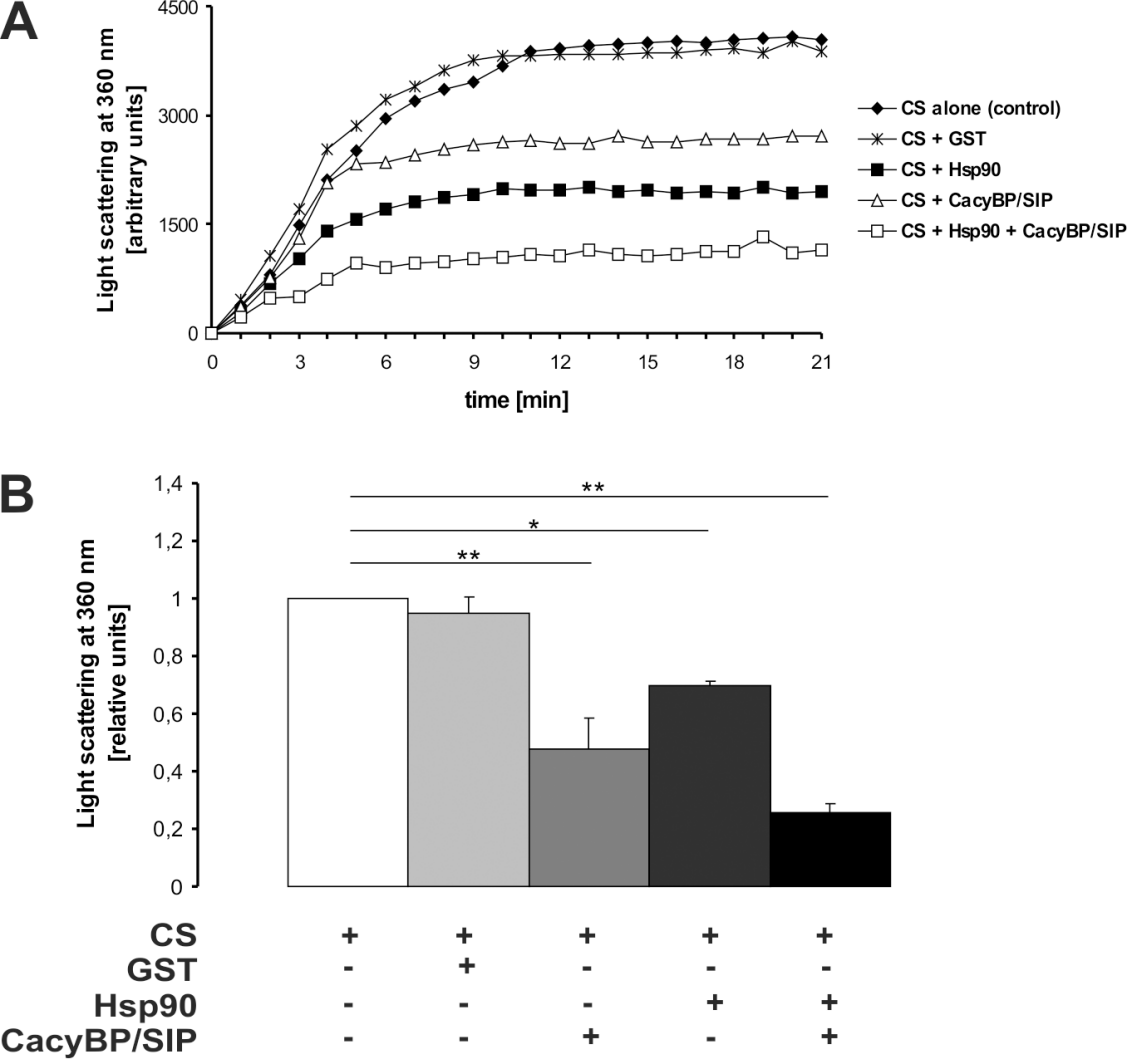
Influence of CacyBP/SIP on heat-induced aggregation of citrate synthase. Aggregation of 0.15 μM citrate synthase (CS) was measured by the increase in optical density due to light scattering (at 360 nm) during incubation at 43°C for 21 min in the absence (control) or presence of GST (negative control), Hsp90, CacyBP/SIP or both Hsp90 and CacyBP/SIP at 0.15 μM final concentration each; **A)** a representative result (out of three performed) is shown as a function of time. **B)** Mean relative levels of CS aggregation in the presence of equimolar concentrations of indicated proteins at the 20-min time point of the aggregation reaction. Data from 3 independent experiments are shown as a mean ± standard error (SEM); * p<0.05, ** p< 0.01.

To check whether CacyBP/SIP exhibits chaperone properties, the ability of this protein to maintain luciferase in a native, folding-competent conformation during thermal inactivation was examined. Recombinant firefly luciferase was thermally denatured alone (control) or in the presence of CacyBP/SIP and Hsp90. Then, renaturation was allowed to proceed and luciferase activity was measured. Data are presented in Fig 7 as percentage of initial luciferase activity. When CacyBP/SIP alone was present, 40% recovery of luciferase activity was observed, as compared to 46% recovery in the presence of Hsp90. When both CacyBP/SIP and Hsp90 were included, the luciferase activity reached 62% of the initial value (Fig 7A). A similar tendency was observed in another approach, in which Hsp40 and Hsp70 were added to each sample directly after denaturation in order to facilitate the renaturation process. In this case, 54%, 70% and nearly 100% of luciferase activity was recovered when, respectively, CacyBP/SIP, Hsp90 or both proteins were present during luciferase inactivation (Fig 7B). Chaperone properties of CacyBP/SIP were also examined in the citrate synthase (CS) aggregation assay. To determine the extent of thermally induced CS aggregation the increase in optical density due to light scattering was measured. As it can be seen in Fig 8, CacyBP/SIP alone as well as in cooperation with Hsp90 inhibits aggregation of this enzyme. In control experiments thermal self-aggregation of the examined proteins (CS, Hsp90, CacyBP/SIP and GST) was checked and it was found that none of these proteins formed aggregates (S2 Fig).

Taken together, the obtained results indicate that CacyBP/SIP together with Hsp90 as well as on its own is able to protect recombinant luciferase from temperature-induced denaturation and to inhibit aggregation of CS. It suggests that the CacyBP/SIP protein exhibits chaperone properties and might be involved in regulation of the Hsp90 chaperone function.

### Phosphatase activity of CacyBP/SIP towards Hsp90

**Fig 9:**
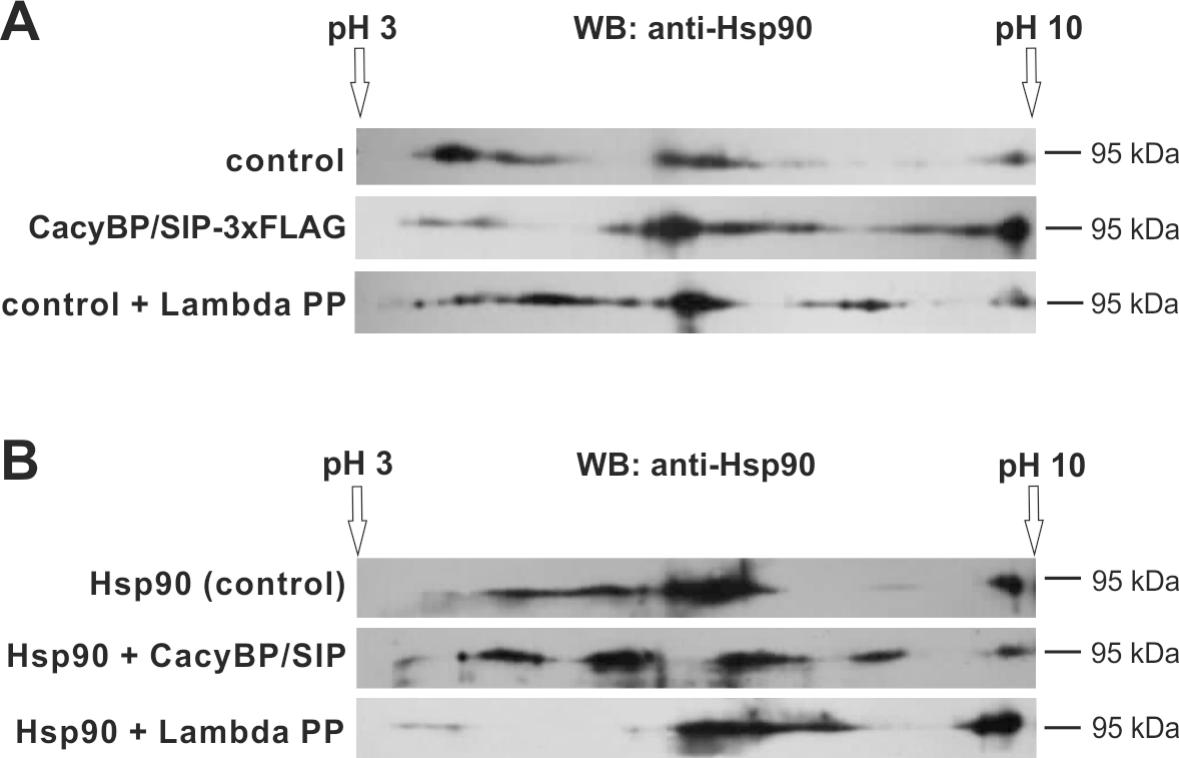
Pattern of spots representing Hsp90 forms with different pI values. **(A)** Pattern of Hsp90 spots in HEp-2 cell lysate. Upper panel: HEp-2 cells co-transfected with plasmids encoding Hsp90β-3xFLAG and 3xFLAG (control). Middle panel: HEp-2 cells co-transfected with plasmids encoding Hsp90β-3xFLAG and CacyBP/SIP-3xFLAG. Bottom panel: HEp-2 cells co-transfected with plasmids encoding Hsp90β-3xFLAG and 3xFLAG (control) but lysate was incubated with Lambda Protein Phosphatase (Lambda PP). (**B)** Pattern of spots corresponding to purified Hsp90. Upper panel: Hsp90 (control). Middle panel: Hsp90 incubated with recombinant CacyBP/SIP. Bottom panel: Hsp90 incubated with Lambda Protein Phosphatase (Lambda PP). A representative Western blot, out of 3 performed after two dimensional (2D) electrophoresis, is shown.

Taking into account that CacyBP/SIP has been shown to serve as a phosphatase for ERK1/2 kinase [20,30], we checked the ability of CacyBP/SIP to dephosphorylate Hsp90. To examine whether CacyBP/SIP might dephosphorylate Hsp90 in the cell, HEp-2 cells were co-transfected with plasmid encoding Hsp90β-3xFLAG and either plasmid encoding 3xFLAG (control) or CacyBP/SIP-3xFLAG. The pattern of Hsp90 forms with different isoelectric points (pI) was analyzed by 2D electrophoresis followed by Western blot (Fig 9A). After expression of CacyBP/SIP-3xFLAG (Fig 9A, middle panel), the protein spots corresponding to Hsp90 forms are shifted in the direction of more basic pH values in comparison to the control (Fig 9A, upper panel). Interestingly, the pattern of Hsp90 forms after incubation of cell lysate with Lambda Protein Phosphatase (Lambda PP) (Fig 9A, bottom panel) is somehow similar to the pattern obtained for the lysate of cells expressing CacyBP/SIP-3xFLAG.

In another approach, purified Hsp90 was incubated alone (control) or in the presence of recombinant CacyBP/SIP or Lambda Protein Phosphatase (Lambda PP) and then analyzed by 2D electrophoresis (Fig 9B). When Lambda Protein Phosphatase (Lambda PP) was present a pronounced shift of Hsp90 forms towards basic pI values could be observed (Fig 9B, bottom panel). Incubation with CacyBP/SIP also resulted in the appearance of more basic Hsp90 forms (Fig 9B, middle panel). Altogether, these results suggest that CacyBP/SIP dephosphorylates Hsp90.

## Discussion

CacyBP/SIP is present in various mammalian cells and tissues [16,31] and seems to play a role in many different cellular processes including cell proliferation and differentiation [19]. CacyBP/SIP is a multi-ligand protein. Among its binding partners there are some S100 proteins, components of E3 ubiquitin ligase (Siah-1 and Skp1), cytoskeletal proteins (tubulin, actin and tropomyosin), ERK1/2 and tau protein. In this work, we have shown that CacyBP/SIP interacts with the Hsp90 molecular chaperone. Since the obtained data show that CacyBP/SIP exhibits chaperone properties and might serve as Hsp90 phosphatase, it can be suggested that CacyBP/SIP is involved in regulation of the Hsp90 chaperone machinery.

A number of observations prompted us to investigate whether CacyBP/SIP is able to bind to Hsp90. The CacyBP/SIP amino acid sequence is similar to that of a known Hsp90 co-chaperone, Sgt1. Interestingly, the highest homology between CacyBP/SIP and Sgt1 is seen in the central (CS) and C-terminal (SGS) domains, which bind Skp1 and S100 proteins, respectively [28,32]. Furthermore, Sgt1 binds Hsp90 through the CS and SGS domains [13,14]. Regarding CacyBP/SIP, preliminary NMR [13] and mass spectrometry [22] experiments provide data suggesting its interaction with Hsp90. Also, a recent study concerning chaperone interaction networks, has considered CacyBP/SIP as a CS domain-containing co-chaperone of Hsp90 [23]. However, direct interaction between these two proteins has not been confirmed. The results presented in this work show for the first time that CacyBP/SIP is a novel Hsp90-interacting partner exhibiting chaperone properties. We have observed binding between CacyBP/SIP and Hsp90 both in the cell and *in vitro* with the use of purified proteins. We have shown that CacyBP/SIP and Hsp90 form complexes localized predominantly in the cytoplasm of HEp-2 cells. Results of luciferase renaturation assay and citrate synthase aggregation assay, methods commonly used for searching for chaperone properties [33,34], have shown that CacyBP/SIP acts as a chaperone.

It is worth to highlight that despite the sequence and structure similarity between Sgt1 and CacyBP/SIP [13,28,32], there are some significant differences in terms of their interaction with Hsp90. Firstly, the competitive ELISA assay with the use of recombinant proteins has revealed that CacyBP/SIP does not compete with Sgt1 for binding to Hsp90. It indicates the existence of distinct binding sites for these two proteins within the Hsp90 molecule. The same phenomenon has been reported previously for plant Sgt1 and another known Hsp90 co-chaperone, p23, which is structurally similar to the CS domain of Sgt1 [35]. Secondly, both CacyBP/SIP and Sgt1 co-immunoprecipitate with full-length Hsp90(α and β)-3xFLAG, whereas only CacyBP/SIP is detected among the proteins associated with FLAG-tagged Hsp90β deletion mutants. According to these results, CacyBP/SIP seems to interact with the middle (M) domain of Hsp90. In contrast, human Sgt1 requires the full-length Hsp90, although plant Sgt1 has been shown to associate with the N-terminal domain of Hsp90 [35,36]. Finally, CacyBP/SIP, but not Sgt1, co-immunoprecipitates with Hsp90β-3xFLAG in lysates obtained from HEp-2 cells treated with radicicol or novobiocin. Radicicol and novobiocin, which bind to the N-terminal or C-terminal domain of Hsp90, respectively, inhibit Hsp90 function by precluding the interaction of Hsp90 with its clients, such as Raf-1 kinase and other co-chaperones [37–39]. Nevertheless, our results show that the CacyBP/SIP-Hsp90 complex is formed both in the absence of the ATP-binding domain and when the Hsp90 ATPase activity is inhibited. According to these findings, we conclude that CacyBP/SIP interacts with Hsp90 in an ATP-independent manner. Moreover, interaction of CacyBP/SIP with Hsp90 deletion mutants that lack the dimerization domain, Hsp90β(N+M) and Hsp90β(M), proves that the binding is mediated by a monomeric middle (M) domain of Hsp90. Thus, although Sgt1 and CacyBP/SIP have similar sequence and structure, they bind to Hsp90 in a distinct manner and, in consequence, might play a different role in the Hsp90 chaperone system.

Apart from co-chaperones, Hsp90 activity is regulated by various post-translational modifications that include phosphorylation, acetylation, S-nitrosylation, oxidation and ubiquitination [9,40]. Among these modifications, phosphorylation of Hsp90 seems to be the most commonly occurring one. It is interesting, that majority of the amino acid residues that undergo phosphorylation are present in the N-terminal and middle part of the Hsp90 molecule [9,41]. This suggests that phosphorylation might regulate ATPase activity and thus determine the binding affinity of some co-chaperones and client proteins. Indeed, phosphorylation of key residues specifically modulates conformational changes of Hsp90 during the ATPase cycle and affects its ability to interact with distinct client proteins. On the other hand, protein phosphatase 5 (PP5/Ppt1), also known as a co-chaperone of Hsp90, dephosphorylates Hsp90 and positively regulates its chaperone activity [42]. In the light on these data, our results demonstrating that CacyBP/SIP changes the pattern of Hsp90 spots with different isoelectric points suggest that CacyBP/SIP might play a significant role in regulation of Hsp90 function through dephosporylation of the chaperone. Since Hsp90 residues influenced by CacyBP/SIP phosphatase are not known at present, future studies may include identification of these residues as well as searching for the physiological effect of Hsp90 dephosphorylation by CacyBP/SIP.

## Conclusion

In this work we have shown that CacyBP/SIP interacts with Hsp90 and exhibits chaperone properties. Immunoprecipitation assay showed that the middle (M) domain of Hsp90 is responsible for CacyBP/SIP binding and the proximity ligation assay (PLA) revealed the presence of CacyBP/SIP-Hsp90 complexes in the cytoplasm of HEp-2 cells. ELISA with the use of purified proteins has shown that Hsp90 interacts directly with CacyBP/SIP and that CacyBP/SIP and Sgt1 do not compete for the binding to Hsp90. CacyBP/SIP was also shown to protect luciferase from thermal denaturation and citrate synthase from aggregation, which suggests that CacyBP/SIP exhibits chaperone properties. As a result of CacyBP/SIP-3xFLAG expression in HEp-2 cells, a shift in spots, representing Hsp90 forms with different isoelectric points, towards more basic pH values was observed. This may indicate that CacyBP/SIP dephosphorylates Hsp90. Altogether, the presented results suggest that CacyBP/SIP is involved in regulation of the Hsp90 chaperone machinery.

## Supporting Information

S1 Fig Control proximity ligation assays (PLA) performed on HEp-2 cells. Antibodies against Hsp90 alone (upper panel). Absence of ligase, a critical reagent for PLA assay (middle panel). Primary antibodies against Hsp90 and against ATP synthase C, a protein which does not interact with Hsp90 (bottom panel). Cell nuclei, stained with DAPI, are in blue. Scale bar is 10 μm.
(TIF)

S2 Fig Measurements of aggregation of citrate synthase (CS), GST, Hsp90 and CacyBP/SIP. Aggregation of these proteins at 43°C (0.15 μM concentration) in a buffer containing 40 mM HEPES, pH 7.5 and 1 mM ATP, was monitored by measurement of optical density at 360 nm during 21 min.
(TIF)

## Abbreviations

BSA: bovine serum albumin
CacyBP/SIP: calcyclin (S100A6) binding protein/Siah-1 interacting protein
co-IP: co-immunoprecipitation
DMEM: Dulbecco’s Modified Eagle’s Medium
DTT: dithiothreitol
GST: glutathione S-transferase
HEp-2: Human Epidermal Carcinoma
pI: isoelectric point
IEF: isoelectrofocusing
PBS: phosphate buffered saline
PAGE: polyacrylamide gel electrophoresis
SDS: sodium dodecyl sulfate
Sgt1: Suppressor of G2 allele of Skp1
TBS: tris buffered saline
2D: two dimensional electrophoresis

## References

[1] Picard D.Heat-shock protein 90, a chaperone for folding and regulation. Cell Mol Life Sci. 2002, 59(10): 1640–1648. 12475174 10.1007/PL00012491 PMC11337538

[2] McClellan AJ, Xia Y, Deutschbauer AM, Davis RW, Gerstein M, Frydman J. Diverse cellular functions of the Hsp90 molecular chaperone uncovered using systems approaches. Cell 2007, 131(1): 121–135. 17923092 10.1016/j.cell.2007.07.036

[3] Koga F, Kihara K, Neckers L. Inhibition of cancer invasion and metastasis by targeting the molecular chaperone heat-shock protein 90. Anticancer Res. 2009, 29(3): 797–807. 19414312

[4] Kamal A, Thao L, Sensintaffar J, Zhang L, Boehm MF, Fritz LC, et al A high-affinity conformation of Hsp90 confers tumour selectivity on Hsp90 inhibitors. Nature 2003, 425(6956): 407–410. 14508491 10.1038/nature01913

[5] Lianos GD, Alexiou GA, Mangano A, Mangano A, Rausei S, Boni L, et al The role of heat shock proteins in cancer. Cancer Lett. 2015, 360(2): 114–118. 10.1016/j.canlet.2015.02.026 25721081

[6] Tatokoro M, Koga F, Yoshida S, Kihara K. Heat shock protein 90 targeting therapy: state of the art and future perspective. EXCLI J. 2015, 14: 48–58. 10.17179/excli2015-586 26600741 PMC4652636

[7] Wandinger SK, Richter K, Buchner J. The Hsp90 chaperone machinery. J Biol Chem. 2008, 283(27): 18473–18477. 10.1074/jbc.R800007200 18442971

[8] Li J, Soroka J, Buchner J. The Hsp90 chaperone machinery: conformational dynamics and regulation by co-chaperones. Biochim Biophys Acta 2012, 823(3): 624–635. 10.1016/j.bbamcr.2011.09.003 21951723

[9] Mollapour M, Neckers L. Post-translational modifications of Hsp90 and their contributions to chaperone regulation. Biochim Biophys Acta 2012, 1823(3): 648–655. 10.1016/j.bbamcr.2011.07.018 21856339 PMC3226900

[10] Prodromou C. The 'active life' of Hsp90 complexes. Biochim Biophys Acta 2012, 1823(3): 614–623. 10.1016/j.bbamcr.2011.07.020 21840346 PMC3793855

[11] Röhl A1, Rohrberg J, Buchner J. The chaperone Hsp90: changing partners for demanding clients. Trends Biochem Sci. 2013, 38(5): 253–262. 10.1016/j.tibs.2013.02.003 23507089

[12] Kitagawa K, Skowyra D, Elledge SJ, Harper JW, Hieter P. SGT1 encodes an essential component of the yeast kinetochore assembly pathway and a novel subunit of the SCF ubiquitin ligase complex. Mol Cell 1999, 4(1): 21–33. 10445024 10.1016/s1097-2765(00)80184-7

[13] Lee YT, Jacob J, Michowski W, Nowotny M, Kuznicki J, Chazin WJ. Human Sgt1 binds HSP90 through the CHORD-Sgt1 domain and not the tetratricopeptide repeat domain. J Biol Chem. 2004, 279(16): 16511–16517. 14761955 10.1074/jbc.M400215200

[14] Spiechowicz M, Zylicz A, Bieganowski P, Kuznicki J, Filipek A. Hsp70 is a new target of Sgt1—an interaction modulated by S100A6. Biochem Biophys Res Commun. 2007, 357(4): 1148–1153. 17466273 10.1016/j.bbrc.2007.04.073

[15] Filipek A, Wojda U. p30, a novel protein target of mouse calcyclin (S100A6). Biochem J. 1996, 320: 585–587 8973570 10.1042/bj3200585 PMC1217969

[16] Filipek A, Kuznicki J. Molecular cloning and expression of a mouse brain cDNA encoding a novel protein target of calcyclin. J Neurochem. 1998, 70(5): 1793–1798. 10.1046/j.1471-4159.1998.70051793.x 9572262

[17] Matsuzawa SI and Reed JC. Siah-1, SIP, and Ebi collaborate in a novel pathway for beta-catenin degradation linked to p53 responses. Mol Cell 2001, 7(5): 915–926. 11389839 10.1016/s1097-2765(01)00242-8

[18] Schneider G, Filipek A. S100A6 binding protein and Siah-1 interacting protein (CacyBP/SIP): spotlight on properties and cellular function. Amino Acids 2011, 41(4): 773–780. 10.1007/s00726-010-0498-2 20182755

[19] Topolska-Woś AM, Chazin WJ, Filipek A. CacyBP/SIP—Structure and variety of functions. Biochim Biophys Acta 2016, 1860(1): 79–85. 10.1016/j.bbagen.2015.10.012 26493724

[20] Kilanczyk E, Filipek S, Filipek A. ERK1/2 is dephosphorylated by a novel phosphatase—CacyBP/SIP. Biochem Biophys Res Commun. 2011, 404(1): 179–183. 10.1016/j.bbrc.2010.11.088 21110948

[21] Wasik U, Schneider G, Mietelska-Porowska A, Mazurkiewicz M, Fabczak H, Weis S, et al Calcyclin binding protein and Siah-1 interacting protein in Alzheimer's disease pathology: neuronal localization and possible function. Neurobiol Aging 2013, 34(5): 1380–1388. 10.1016/j.neurobiolaging.2012.11.007 23260124

[22] Gano JJ, Simon JA. A proteomic investigation of ligand-dependent HSP90 complexes reveals CHORDC1 as a novel ADP-dependent HSP90-interacting protein. Mol Cell Proteomics 2010, 9(2): 255–270. 10.1074/mcp.M900261-MCP200 19875381 PMC2830838

[23] Taipale M, Tucker G, Peng J, Krykbaeva I, Lin ZY, Larsen B, et al A quantitative chaperone interaction network reveals the architecture of cellular protein homeostasis pathways. Cell 2014, 158(2): 434–448. 10.1016/j.cell.2014.05.039 25036637 PMC4104544

[24] Bradford MM. A rapid and sensitive method for the quantitation of microgram quantities of protein utilizing the principle of protein-dye binding. Anal Biochem. 1976, 72:248–254. 942051 10.1016/0003-2697(76)90527-3

[25] Laemmli UK. Cleavage of structural proteins during the assembly of the head of bacteriophage T4. Nature 1970, 227(5259): 680–685. 5432063 10.1038/227680a0

[26] Zurawska A, Urbanski J, Matuliene J, Baraniak J, Klejman MP, Filipek S, et al Mutations that increase both Hsp90 ATPase activity in vitro and Hsp90 drug resistance in vivo. Biochim Biophys Acta 2010, 1803(5): 575–583. 10.1016/j.bbamcr.2010.03.002 20226818

[27] Filipek A, Jastrzebska B, Nowotny M, Kuznicki J. CacyBP/SIP, a calcyclin and Siah-1-interacting protein, binds EF-hand proteins of the S100 family. J Biol Chem. 2002, 277(32): 28848–28852. 12042313 10.1074/jbc.M203602200

[28] Nowotny M, Spiechowicz M, Jastrzebska B, Filipek A, Kitagawa K, Kuznicki J. Calcium-regulated interaction of Sgt1 with S100A6 (calcyclin) and other S100 proteins. J Biol Chem. 2003, 278(29): 26923–26928. 12746458 10.1074/jbc.M211518200

[29] Buchner J, Grallert H, Jakob U. Analysis of chaperone function using citrate synthase as nonnative substrate protein. Methods Enzymol. 1998, 290: 323–338. 9534173 10.1016/s0076-6879(98)90029-5

[30] Topolska-Woś AM, Shell SM, Kilańczyk E, Szczepanowski RH, Chazin WJ, Filipek A. Dimerization and phosphatase activity of calcyclin-binding protein/Siah-1 interacting protein: the influence of oxidative stress. FASEB J. 2015, 29(5): 1711–1724. 10.1096/fj.14-264770 25609429 PMC4415008

[31] Zhai H, Shi Y, Jin H, Li Y, Lu Y, Chen X, et al Expression of Calcyclin-binding Protein/Siah-1 Interacting Protein in Normal and Malignant Human Tissues: An Immunohistochemical Survey. J Histochem Cytochem. 2008, 56(8): 765–772. 10.1369/jhc.2008.950519 18443365 PMC2443607

[32] Bhattacharya S, Lee YT, Michowski W, Jastrzebska B, Filipek A, Kuznicki J, et al The modular structure of SIP facilitates its role in stabilizing multiprotein assemblies. Biochemistry 2005, 44(27): 9462–9471. 15996101 10.1021/bi0502689

[33] Zabka M, Leśniak W, Prus W, Kuźnicki J, Filipek A. Sgt1 has co-chaperone properties and is up-regulated by heat shock. Biochem Biophys Res Commun. 2008, 370(1):179–183. 10.1016/j.bbrc.2008.03.055 18358234

[34] Lu Z, Cyr DM. Protein folding activity of Hsp70 is modified differentially by the hsp40 co-chaperones Sis1 and Ydj1. J Biol Chem. 1998, 273(43): 27824–27830. 9774392 10.1074/jbc.273.43.27824

[35] Kadota Y, Amigues B, Ducassou L, Madaoui H, Ochsenbein F, Guerois R, et al Structural and functional analysis of SGT1-HSP90 core complex required for innate immunity in plants. EMBO Rep. 2008, 9(12): 1209–1215. 10.1038/embor.2008.185 18833289 PMC2570500

[36] Kadota Y, Shirasu K, Guerois R. NLR sensors meet at the SGT1-HSP90 crossroad. Trends Biochem Sci. 2010, 35(4): 199–207. 10.1016/j.tibs.2009.12.005 20096590

[37] Schulte TW, Akinaga S, Soga S, Sullivan W, Stensgard B, Toft D, et al Antibiotic radicicol binds to the N-terminal domain of Hsp90 and shares important biologic activities with geldanamycin. Cell Stress Chaperones 1998, 3(2): 100–108. 9672245 10.1379/1466-1268(1998)003<0100:arbttn>2.3.co;2 PMC312953

[38] Marcu MG, Chadli A, Bouhouche I, Catelli M, Neckers LM. The heat shock protein 90 antagonist novobiocin interacts with a previously unrecognized ATP-binding domain in the carboxyl terminus of the chaperone. J Biol Chem. 2000, 275(47): 37181–37186. 10945979 10.1074/jbc.M003701200

[39] Allan RK, Mok D, Ward BK, Ratajczak T. Modulation of chaperone function and cochaperone interaction by novobiocin in the C-terminal domain of Hsp90: evidence that coumarin antibiotics disrupt Hsp90 dimerization. J Biol Chem. 2006, 281(11): 7161–7171. 16421106 10.1074/jbc.M512406200

[40] Scroggins BT, Neckers L. Post-translational modification of heat-shock protein 90: impact on chaperone function. Expert Opin Drug Discov. 2007, 2(10): 1403–1414. 10.1517/17460441.2.10.1403 23484535

[41] Cloutier P, Coulombe B. Regulation of molecular chaperones through post-translational modifications: decrypting the chaperone code. Biochim Biophys Acta 2013, 1829(5): 443–454. 10.1016/j.bbagrm.2013.02.010 23459247 PMC4492711

[42] Wandinger SK, Suhre MH, Wegele H, Buchner J. The phosphatase Ppt1 is a dedicated regulator of the molecular chaperone Hsp90. EMBO J. 2006, 25(2): 367–376. 16407978 10.1038/sj.emboj.7600930 PMC1383513

