# Supplementary figures and images for: Calcyclin Binding Protein/Siah-1 Interacting Protein Is a Hsp90 Binding Chaperone
Source: PLoS One. 2016 Jun 1;11(6):e0156507. doi: 10.1371/journal.pone.0156507 (PMC4889068; doi:10.1371/journal.pone.0156507)

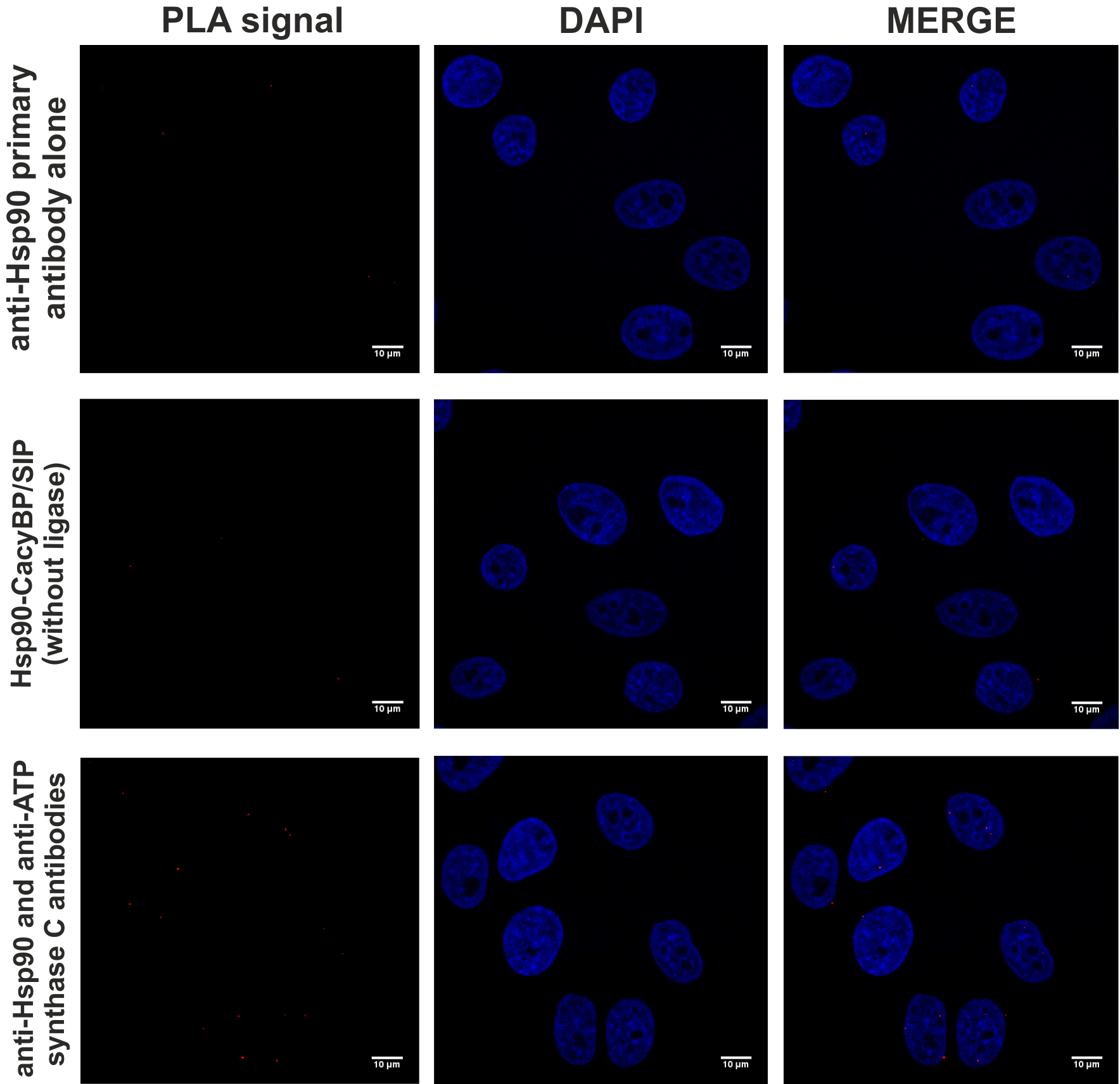

Supplement: S1 Fig — Antibodies against Hsp90 alone (upper panel). Absence of ligase, a critical reagent for PLA assay (middle panel). Primary antibodies against Hsp90 and against ATP synthase C, a protein which does not interact with Hsp90 (bottom panel). Cell nuclei, stained with DAPI, are in blue. Scale bar is 10 μm. (TIF) [file pone.0156507.s001.tif]

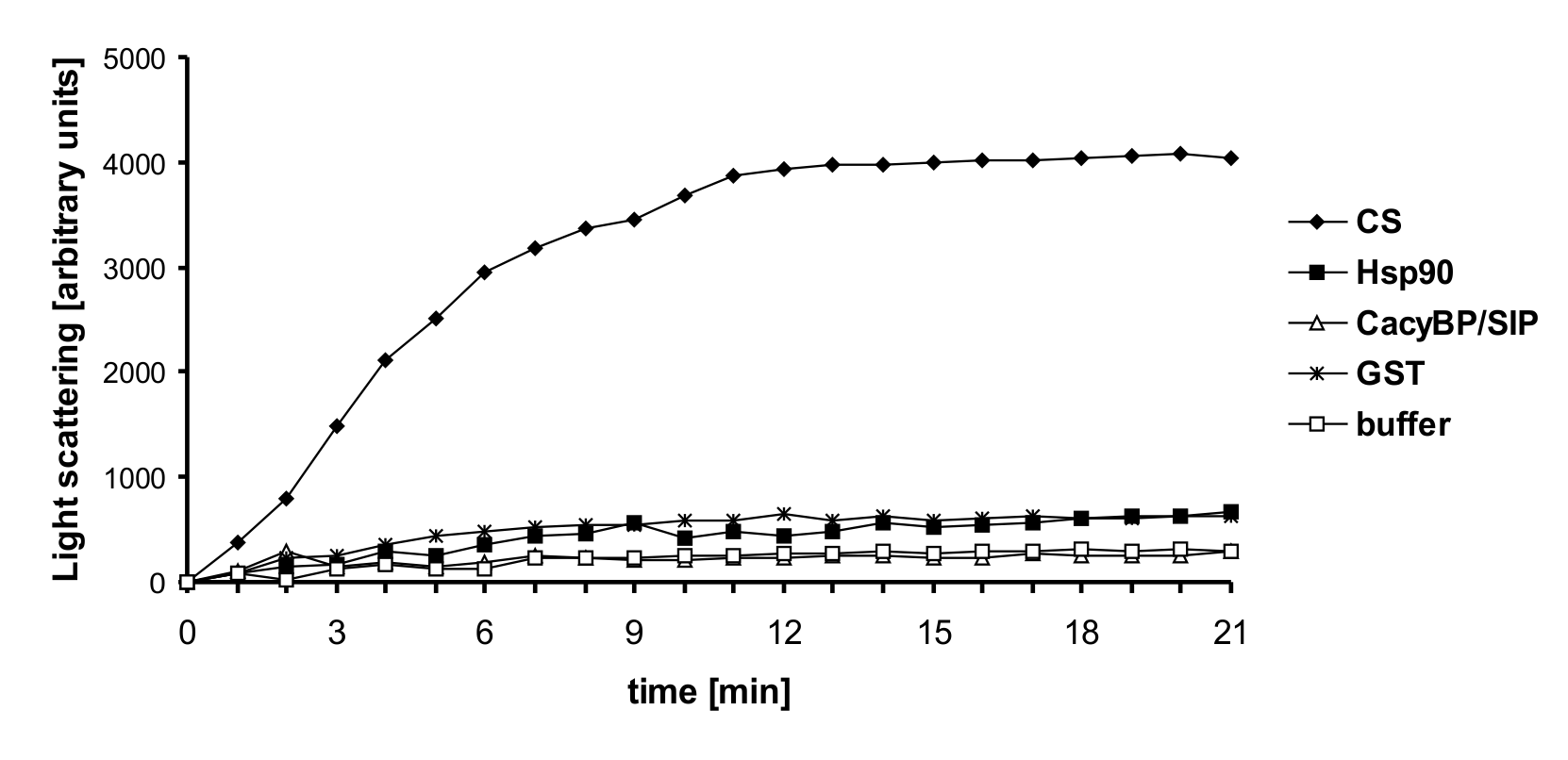

Supplement: S2 Fig — Aggregation of these proteins at 43°C (0.15 μM concentration) in a buffer containing 40 mM HEPES, pH 7.5 and 1 mM ATP, was monitored by measurement of optical density at 360 nm during 21 min. (TIF) [file pone.0156507.s002.tif]
